# Microfluidics and Organ-on-a-Chip for Disease Modeling and Drug Screening

**DOI:** 10.3390/bios14020086

**Published:** 2024-02-04

**Authors:** Rohollah Nasiri, Yangzhi Zhu, Natan Roberto de Barros

**Affiliations:** 1 Science for Life Laboratory, Division of Nanobiotechnology, Department of Protein Science, KTH Royal Institute of Technology, 17165 Solna, Sweden; 2 Terasaki Institute for Biomedical Innovation, Los Angeles, CA 91367, USA; yzhu@terasaki.org (Y.Z.); nbarros@terasaki.org (N.R.d.B.)

The convergence of microfluidics and organ-on-a-chip (OoC) technologies has revolutionized our ability to create advanced *in vitro* models that recapitulate complex physiological processes. Designed to replicate the key features of human organs outside the body, these platforms are increasingly employed to investigate organ functionality for applications such as disease modeling, drug testing, and personalized medicine. Moreover, these *in vitro* models have the potential to expedite drug development by reducing, replacing, or refining animal models. This editorial highlights the key contributions from a Special Issue on this subject, shedding light on the progress made in the field of microphysiological systems, particularly in the areas of nephrotoxicity assessment, 3D bioprinting of liver matrix hydrogel, metabolic assessment of astrocytes, brain-on-a-chip models, gut-on-a-chip technology, biosensors for thrombin detection, and an academic viewpoint on organ-on-a-chip technology.

We are pleased with the quality of the articles submitted to our Special Issue entitled “Microfluidics and Organ-on-a-Chip for Disease Modeling and Drug Screening”. This Special Issue collected three research articles, three review articles, and one perspective article that address the recent advances and insights in microphysiological systems from scholars in various countries (including the United States, Sweden, Norway, the Netherlands, China, Iran, and Australia) and from nominated universities/institutions (such as KTH Royal Institute of Technology, Peking University, Eindhoven University of Technology, Griffith University, The University of Queensland, Iran University of Science and Technology, NouBio Inc., the University of Valencia, and the University of Oslo). In this editorial, we provide a brief introduction to these published articles and welcome readers to refer to these publications and their associated references for more details on the relevant topics.

In a paper by Jing et al. (Contribution 1), entitled “Functional Evaluation and Nephrotoxicity Assessment of Human Renal Proximal Tubule Cells on a Chip”, the authors introduce an innovative high-throughput human renal proximal tubule model based on an integrated biomimetic array chip (iBAC). The study emphasizes the importance of accurate transporter expression and epithelial polarization for nephrotoxicity prediction. The developed renal proximal tubule model on the chip demonstrates improved barrier function, transporter activity, and apical–basolateral polarization, showcasing its potential as an effective nephrotoxicity screening toolkit.

In a paper from Prof. Aman Russom’s group, entitled “3D Bioprinting of Multi-Material Decellularized Liver Matrix Hydrogel”, Khati et al. (Contribution 2) propose a bioink to address the challenge of poor mechanical properties in the decellularized extracellular matrix (dECM). This paper presents a multi-material decellularized liver matrix (dLM) bioink reinforced with gelatin and polyethylene glycol. The bioink facilitates extrusion-based bioprinting at physiological temperatures, offering enhanced rheology, extrudability, and mechanical stability. The resulting heavily crosslinked structure holds promise for extended 3D-printing procedures with maintained cell viability and liver-specific functions.

In a research article from Prof. Anna Herland’s group, the authors compare the metabolic turnover of glucose and lactate in astrocytes derived from human induced pluripotent stem cell (hiPSC) astrocytes (hiAstrocytes) and primary human astrocytes using a commercial microfluidic glucose/lactate biosensor. This study provides valuable insights into scalable and functional sources for *in vitro* studies of astrocyte function. The results elaborate on the use of hiAstrocytes and flow-based biosensors for metabolic studies, highlighting their potential in understanding astrocyte function and the benefits of using these sensors for advanced *in vitro* neural systems (Contribution 3).

Considering microphysiological systems for the brain, in a review article by Prof. Regina Luttge’s group, the authors emphasize the importance of microenvironments in brain-on-a-chip models for accurately emulating the unique organization of the human brain structure. They discuss the significance of regional stiffness gradients, mechanical properties, and advanced *in vitro* platforms. Their paper provides a comprehensive overview of the recent developments in brain modeling efforts, incorporating brainoids, brain-on-a-chip (BoC) platform technology, 3D-printed gels, and other engineered guidance features. The conclusions drawn from their study aim to advance instructive microenvironments for BoCs and enhance our understanding of the brain’s cellular functions in both healthy and diseased states (Contribution 4).

In another review article, Thomas (Contribution 5) discuss the fundamentals and challenges of microfluidic gut-on-a-chip (GOC) technology. Their review encompasses the physiology of the human gut, the engineering approaches of GOC models, materials and fabrication, cell types, stimuli, and gut microbiota. Their paper not only discusses current applications but also addresses the challenges, possible solutions, and prospects for GOC models and technology. This comprehensive review provides a holistic view of the advancements and limitations in the field.

As biosensors are a key addition to the field of organ-on-a-chip for the real-time monitoring of tissue function, in a review article led by Ahadian (Contribution 6), the authors summarize the recent progress in the development of biosensors for thrombin detection, providing great insights for integrating these sensors into heart-on-a-chip models. This paper delves into various biosensors designed for measuring thrombin levels. The paper introduces the concept of aptasensors, highlighting the use of DNA or RNA aptamers for highly selective and specific detection of thrombin. It also provides an update on biosensors, summarizing their construction methods, compositions, performance, benefits, and limitations. This information contributes to the development of effective methods for sensitive and specific thrombin detection in biological analysis and clinical diagnosis.

Finally, in a perspective paper by Busek (Contribution 7), OoC systems are evaluated from an academic end-user perspective, considering usability, complexity, and robustness. Drawing insights from a survey of 187 peers in 35 countries, the paper highlights the strengths and limitations of current commercial OoC platforms and self-made systems. It emphasizes the need for next-generation OoCs that balance robustness and complexity, providing a guide for researchers in the field and encouraging further advancements.

In conclusion, the diverse range of contributions in this Special Issue showcases the remarkable progress in microfluidics and organ-on-a-chip technologies. These advancements hold great promise for advancing disease modeling, drug screening, and our overall understanding of complex biological systems. As we navigate the future of biomedical research, the collaboration between researchers in different research areas, including engineers, biologists, clinicians, and material scientists, will continue to drive innovation in these transformative fields. Furthermore, as we envision the future trajectory of organ-on-a-chip development, the potential integration of artificial intelligence emerges as a promising avenue, fostering unprecedented advancements in precision medicine and drug discovery within this transformative field.

